# Threat affects risk preferences in movement decision making

**DOI:** 10.3389/fnbeh.2015.00150

**Published:** 2015-06-09

**Authors:** Megan K. O'Brien, Alaa A. Ahmed

**Affiliations:** Neuromechanics Laboratory, Department of Integrative Physiology, University of ColoradoBoulder, CO, USA

**Keywords:** neuroeconomics, sensorimotor control, risk-sensitivity, postural threat, prospect theory

## Abstract

Emotional states such as sadness, anger, and threat have been shown to play a critical role in decision-making processes. Here we addressed the question of whether risk preferences are influenced by postural threat and whether this influence generalizes across motor tasks. We examined risk attitudes in the context of arm-reaching (ARM) and whole-body (WB) leaning movements, expecting that increased postural threat would lead to proportionally similar changes in risk-sensitivity for each motor task. Healthy young adults were shown a series of two-alternative forced-choice lotteries, where they were asked to choose between a riskier lottery and a safer lottery on each trial. Our lotteries consisted of different monetary rewards and target sizes. Subjects performed each choice task at ground level and atop an elevated platform. In the presence of this postural threat, increased physiological arousal was correlated with decreased movement variability. To determine risk-sensitivity, we quantified the frequency with which a subject chose the riskier lottery and fit lottery responses to a choice model based on cumulative prospect theory (CPT). Subjects exhibited idiosyncratic changes in risk-sensitivity between motor tasks and between elevations. However, we found that overweighting of small probabilities increased with postural threat in the WB task, indicating a more cautious, risk-averse strategy is ascribed to the possibility of a fall. Subjects were also more risk-seeking in the WB movements than in ARM at low elevation; this behavior does not seem to derive from consistent distortions in utility or probability representations but may be explained by subjects' inaccurate estimation of their own motor variability. Overall, our findings suggest that implicit threat can modify risk attitudes in the motor domain, and the threat may induce risk-aversion in salient movement tasks.

## Introduction

Recent work suggests that an individual's emotional state can dictate decision making. For instance, affective reactions to a stimulus, either positive or negative, can alter our subjective interpretations of perceived risks and benefits, thereby impacting our cognitive processes and choices (Slovic, 1987; Finucane et al., 2000; Loewenstein et al., 2001; Slovic et al., 2002, 2004; Loewenstein and Lerner, 2003). Stress—such as one might experience before delivering a public speech or while immersing one's hand in icy water—has specifically been shown to modulate risk-sensitivity in economic decision making. Stressed participants reduce their risk-taking behavior in the face of potential monetary losses (Pabst et al., 2013), increase risk-taking for potential monetary gains (Buckert et al., 2014), or vise versa (Porcelli and Delgado, 2009).

How does emotion affect our movements? The aforementioned connection between stress and decision making highlights an intriguing yet underappreciated finding in whole-body (WB) movement control. Particularly relevant stressors in a movement domain are environments of postural threat, which increase the consequences or probability of unsuccessful movement, either implicitly or explicitly. Even when there is a minimal increase in postural threat (such as standing on a balance board), individuals significantly alter their movement strategies (Huang and Ahmed, 2011; Manista and Ahmed, 2012; Pienciak-Siewert et al., 2014). Elevation is a common method of increasing postural threat that has a marked effect of on movement strategies in WB movement control. Standing on an elevated platform leads to increased measures of physiological arousal, which indicates greater levels of anxiety (Ashcroft et al., 1991; Brown et al., 2002, 2006). And when asked to walk or stand on an elevated platform, both young and old adults reduce the velocity and extent of their postural movements (Carpenter et al., 1999, 2001, 2006; Adkin et al., 2000, 2002, 2008; Brown et al., 2006; Davis et al., 2009; Lamarche et al., 2009). Importantly, it appears that these movement modifications are a function of the amount of threat, wherein the central nervous system increasingly tightens its control of posture with elevation (Adkin et al., 2000). While changes in movement control on elevated platforms cannot be explained by changes in biomechanical capacity, they can be explained by risk-sensitive changes in decision making. Framing WB movement in neuroeconomic terms suggests that these movement-based changes can result from the feelings of threat associated with standing on an elevated platform. For example, placing higher subjective value on the consequences of a fall or assigning more weight to the probability of a fall should result in more cautious movement decisions. Such risk-sensitive responses would explain the previously observed reductions in postural excursion and velocity. If changes in movement control on elevated platforms result from the feelings of threat experienced while standing on the platform, then it is feasible that these emotions will influence risk-sensitive behavior in motor-based decision tasks as well.

Risk-sensitivity in movement has recently been explored using a variety of behavioral and computational approaches, particularly for arm-reaching (ARM) tasks. In discrete endpoint planning, Trommershauser et al. (2003a,b) found subjects were nearly able to maximize expected gain during a pointing task (suggesting risk-neutrality), whereas Wu et al. (2009, 2011) observed distortions in motor rewards and probabilities using cumulative prospect theory (CPT) (suggesting risk-sensitivity). Evidence of risk-sensitivity has also appeared during target searches with a Bayesian integration model (Grau-Moya et al., 2012), in sensitivity to effort with the mean-variance trade-off (Nagengast et al., 2011), and in sensitivity to error with a motor adaptation model (Trent and Ahmed, 2013). Previously, we investigated risk-sensitivity in ARM and WB movements using a continuous movement paradigm (O'Brien and Ahmed, 2013). Subjects maneuvered a cursor as close to the edge of a virtual cliff as possible and received a point score based on their performance. We found that in both movement tasks, subjects moved closer to the cliff edge than predicted by a risk-neutral model of movement planning, suggesting risk-seeking behavior. We also saw greater risk-seeking behavior in the WB movement than in arm-reaching; however, the cause of such behavior was unclear. A follow-up study intimated that differences in risk-sensitivity between the two tasks emerged from the movements themselves rather than from the sitting and standing postures (O'Brien and Ahmed, 2014). In the present experiment, we specifically investigate whether risk-seeking behavior in movement might have resulted from (i) an inappropriate estimation of sensorimotor variability or (ii) a distorted weighting of point rewards and penalties, by probing subjects' subjective valuation of probability and utility (reward) during a movement task.

The main objective of this study was to examine the influence of postural threat on risk preferences during movement decision making and to assess whether this influence generalizes across different motor tasks. Subjects were asked to choose between risky lotteries in the context of two motor tasks: ARM and WB leaning. They completed each task at ground level and atop an elevated platform. Since individuals reduce the velocity and extent of their movements under postural threat, we expected that increasing postural threat in the form of elevation (low vs. high) would lead to increased risk-aversion. This risk-aversion could manifest itself as decreased subjective valuation of movement rewards or as increased weighting of the probability of a fall. We also expected to see proportionally similar changes for both motor tasks; that is, if an individual became more risk-averse in the ARM task at high elevation, that person would be equally risk-averse in the WB task at high elevation. Findings of this study are pertinent to the analysis of the influence of emotional state on movement decisions under risk. Our results will help determine whether there are generalizable principles such that movement decision-making in various emotional states can be predicted and trained across motor tasks.

## Materials and methods

### Subjects

Twenty right-handed, healthy subjects (13 females, 7 males; mean age, 23.1 ± 2.8 years) participated in this experiment, performing two motor lottery series in both the low-threat and high-threat conditions. Fifteen of the 20 subjects repeated a lottery series during the experiment, allowing us to assess within-subject choice consistency. Thirteen of the 20 subjects were part of a broader study examining the influence of threat on non-motor and motor tasks (O'Brien and Ahmed, 2014). All participants provided informed consent, and the experimental protocol (12-0458) was approved by the Institutional Review Board of the University of Colorado Boulder in accordance with federal regulations, university policies, and ethical standards regarding human subject research.

### Experimental protocol

Subjects performed two motor choice tasks: one for seated arm-reaching (ARM) and another for standing whole-body leaning movements (WB). In each task, subjects were asked to choose between two lotteries, where each lottery has a different monetary reward and probability of winning that reward. Rather than offering explicit probabilities for these lotteries (e.g., 50% chance of winning the reward), we gave subjects implicit probabilities in the form of rectangular targets with variable widths (e.g., such that a subject had a 50% chance of hitting the target in a given motor task), wherein hitting the target results in winning the reward.

Subjects underwent a testing session in both motor choice tasks under two threat conditions: low elevation and high elevation. In the low elevation condition (Low), subjects chose between risky lotteries at ground level, either sitting in a chair for the ARM task or standing at the edge of a forceplate for the WB task. In the high elevation condition (High), subjects sat in the same chair or stood on the same forceplate at the edge of an elevated platform, 0.8 m off the ground. The height of this elevated platform is approximately the average height at which young adults perceived they would not be able to use a step down strategy to descend from an elevated surface (Brown and Frank, 1997). When standing in either elevation condition, subjects were secured in a harness and fall protection system that could arrest a fall before the subjects' knees touched the platform. However, to maintain perceptions of postural threat in the presence of this added safety, there was enough slack to the harness to allow subjects to move without restraint, and they were not allowed to voluntarily explore the competence of the fall protection system.

**Figure 1:**
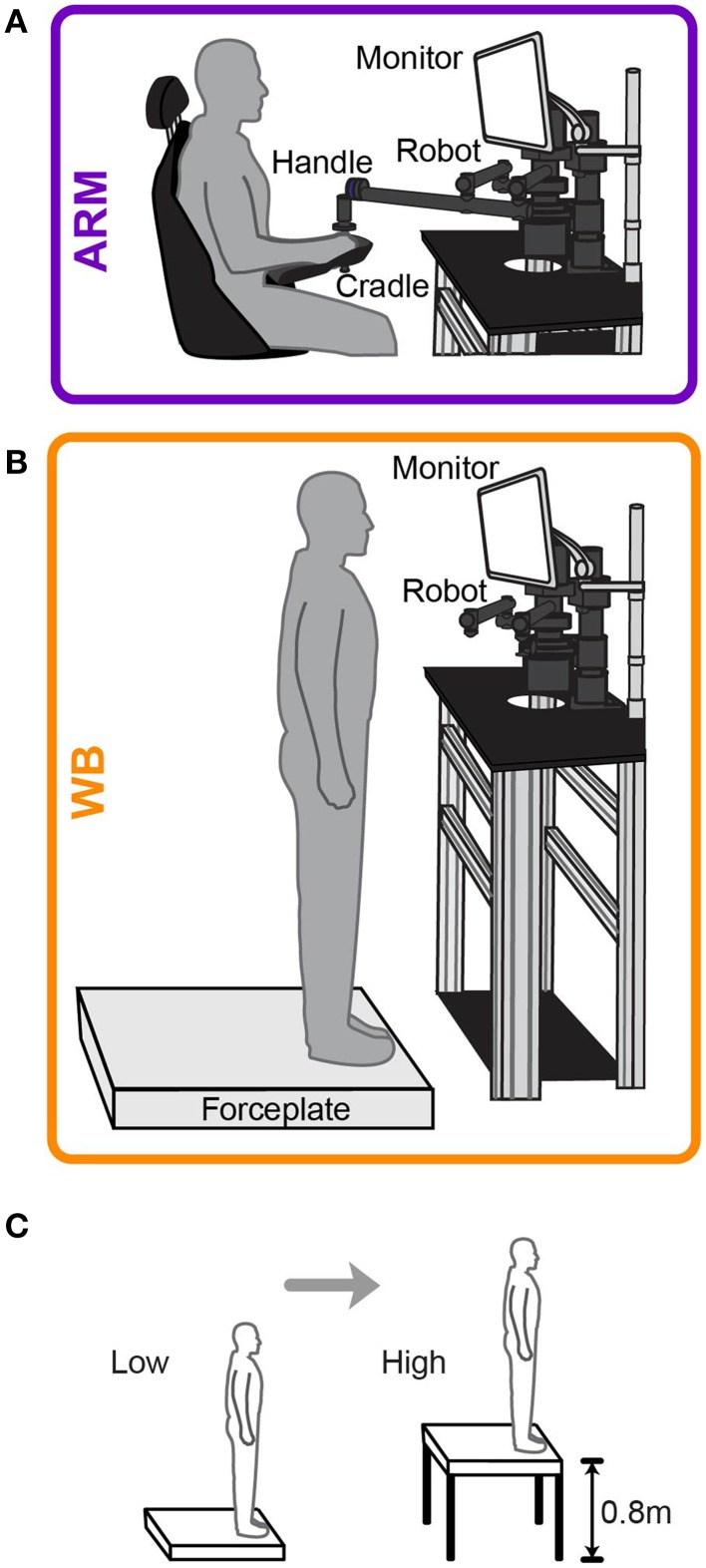
**Experimental setup for motor tasks**. Schematic of **(A)** arm-reaching task (ARM) and **(B)** whole-body (WB) movement task. Subjects executed out-and-back movements to a target during training and realization of choices. **(C)** Subjects performed all movement and choice tasks at ground level (Low elevation) and then at the edge of a 0.8 m platform (High elevation).

Prior to testing at each elevation, subjects were given the opportunity to practice actual movements in a training session. In the ARM task, subjects used their dominant arm to grasp the handle of a robotic manipulandum (Interactive Motion Technologies Shoulder-Elbow Robot 2) and move a cursor from a starting region to a target that was 12 cm directly in front of them. In the WB task, subjects stood on a forceplate (AMTI Dual-Top AccuSway, which is 4.5 cm in height) and used their center of pressure (COP) to move the cursor from a starting region to a target that was 6 cm directly in front of them. Figures 1A,B illustrate the physical configuration for the ARM and WB tasks, respectively. The cursor was a yellow circle with a radius of 0.25 cm and a red vertical line through it to mark the center. The target was a thin, white horizontal line traversing the computer screen and a small, white vertical line at its center. Subjects were instructed to make a quick out-and-back movement from the starting region to the target, trying to move as straight as possible to hit the center of the target with the center of the cursor. However, the horizontal position of the cursor was obscured from the time the cursor left the starting region until they crossed the target line. Visual feedback of the cursor's horizontal position at the target distance was shown after each trial, supplying information about their error relative to the center of the target. We encouraged subjects to hit the target within 750–850 ms by providing feedback about their movement time after each trial. During this feedback, the target flashed green if they moved too quickly, gray if they moved too slowly, and yellow with an auditory tone if they hit the target within the given time window. Subjects performed 100 trials in both ARM and WB tasks. We found that 100 trials were more than sufficient for participants to reach asymptotic levels of performance in both the ARM and WB tasks, requiring approximately 20–40 trials to do so. We explicitly told subjects to pay attention to their performance throughout training, noting how close their cursor was to the target center across trials. We explained that having some idea of their accuracy would help them make decisions about relative target sizes during the choice-based testing session.

During testing, subjects performed the ARM and WB lottery tasks in a randomized order at each elevation, counterbalanced across the two tasks. They completed both choice tasks at low elevation before performing them at high elevation (Figure 1C). It has been previously shown that increasing elevation results in more pronounced changes to postural control variables than decreasing elevation (Adkin et al., 2000). In always presenting the Low elevation condition first, we intended to capitalize on these order effects to maximize potential changes in risk-sensitivity due to threat. Ideally, we would test and re-test as many conditions as possible to examine consistency of choices over the different conditions. However, due to the overall length of the experiment and to minimize mental fatigue, we had subjects repeat one of these tasks, which we still used to examine choice consistency. We selected the WB Low task as the condition to be repeated. Fifteen of the 20 subjects performed this repeated condition, which was included in the randomized conditions at Low elevation. Lotteries were displayed on a computer monitor in front of the subject. In the testing phase of the experiment, subjects simply chose between pairs of lotteries, with every choice completing a single trial. Subjects performed 72 choice trials for each motor task. Within each trial, lottery information was shown for 4 s; the lotteries then disappeared and subjects were given 2 s to select their preferred lottery.

After completing the training and choice tasks at both elevations, subjects participated in a realization of choices phase, where we randomly selected one trial from each task, and the subject “played” their choice on that trial for real money. Playing a choice was similar to the training tasks; however, rather than showing a thin target line, subjects saw the specific target and monetary reward they chose on the selected trial. Subjects rapidly moved the same cursor to the chosen target; if the cursor hit the target, the subject won the reward. Since movement control is inherently variable, there was always a probability that a subject would miss the target (thereby receiving no reward) in these motor tasks. Subjects were aware in advance that a random selection of trials would be played to encourage them to make decisions based on what they would do in a real-life scenario.

### Lottery design

Construction of lottery pairs is based on the design of Wu et al. (2009) and follows what we implemented in O'Brien and Ahmed (2014). Subjects were asked to choose between two lotteries (A and B), each of which had a different monetary reward ($*y* and $*z*) and probability of winning that reward (*p* and *q*). Let us formulate these lotteries as A($*y, p*) and B($*z, q*). For every trial, there was one “safer” lottery and one “riskier” lottery, classified based on the variance of each lottery. We consider the lottery with a higher variance to be the riskier option.

(1) $$\begin{array}{l} \text{Var}[A]=py^{2}(1-p)\\ \text{Var}\left[B\right]=qz^{2}\left(1-q\right) \end{array}$$

**Figure 2:**
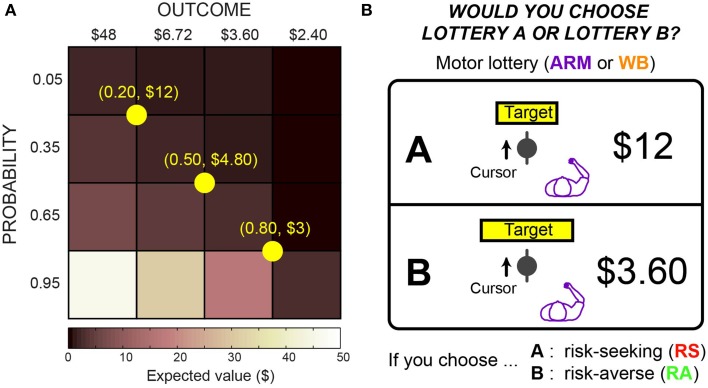
**Motor lotteries. (A)** Presentation of a motor lottery included a target with some width and a monetary reward for hitting that target. Arrow indicates desired direction of cursor movement. Horizontal cursor position at target determines whether the subject would win the reward. **(B)** Lotteries were constructed using a 4 × 4 outcome-probability matrix, where each block is paired with each reference lottery (shown in yellow).

Lottery pairs were presented in three blocks of 24 trials, for a total of 72 trials per task. Each lottery pair consisted of a reference lottery and a varying lottery. The reference lottery was fixed within a block, whereas the varying lottery changed on each trial. We used a 4 × 4 outcome-probability matrix to construct the lottery pairs, as shown in Figure 2A. All reference lotteries had the same expected value. For the varying lottery, there were four possible monetary outcomes ranging from $2.40 to $48, and there were four possible probabilities ranging from 0.05 to 0.95. The diagonal elements of the matrix had nearly the same expected value and were shown three times per block, while the remaining off-diagonal elements were shown once per block. For each subject and task, we randomized the order of the blocks and the order of the varying lotteries within each block.

An example lottery pair for the ARM choice task is shown in Figure 2B. In both motor tasks, subjects were shown monetary rewards and targets of varying widths, but they were not told which lottery was riskier or safer on any given trial. We constructed the target sizes to correspond to certain probabilities of hitting the target. We measured subjects' mediolateral endpoint variability during the ARM and WB training tasks, and we used these variabilities to construct motor lotteries that are equivalent to economic lotteries with explicit probabilities.

### Variability testing and motor lotteries

Prior to the testing phase, subjects had the opportunity to perform practice movements for both the ARM and WB tasks. They performed 100 trials at a prescribed time (750–850 ms) to a thin target line, receiving visual feedback regarding their mediolateral proximity to the center of the target. The target line was located at a single distance for all trials within each movement task (12 cm for ARM and 6 cm for WB). In the realization of choices phase, the target was located at these same distances.

We used the mediolateral standard deviation of the cursor at the target line, σ, to construct the motor lottery target sizes for testing. That is, we adjusted the width of the target during testing so that the subject's probability of hitting the target had an approximate value. From Wu et al. (2011), the relationship between the probability of hitting a target, *p*_target_, and the width of a target for motor lottery, *w*, is:

(2) $$p_{\text{target}}=\int_{x_{0}-0.5w}^{x_{0}+0.5w}\frac{1}{\sqrt{2\pi \sigma^{2}}}e^{{\left(x-x_{0}\right)}^{2}/2\sigma^{2}}dx,$$

where *x* is the horizontal axis, and *x*_0_ is the center of the target.

### Measures of risk-sensitivity

We employed CPT to estimate subject-specific distortions in the utilities and probabilities associated with our lotteries. In CPT, risk-sensitivity can be explained by a distortion in Equation (1) the utility/value function or Equation (2) the probability weighting function (Tversky and Kahneman, 1992). For our lottery task, we employ CPT to model the subjective value function of monetary rewards *v*(*O*) and probability weighting *w*(*P*) as:

(3a) $$v\left(O\right)\;=O^{\alpha },\;O\geq 0$$

(3b) $$w\left(P\right)=\exp\left[-{\left(-\ln\left(P\right)\right)}^{\gamma }\right],\;0<P<1$$

Parameters for utility and probability weightings are α and γ, respectively. Distortions in utility and probability (α, γ ≠ 1) characterize risk-sensitive behavior, with α < 1 and γ < 1 indicative of risk-aversion and overweighting of small probabilities, respectively. Conversely, α > 1 and γ > 1 are indicative of risk-seeking behavior and underweighting small probabilities, respectively.

The cumulative prospects of the two lotteries, A($*y, p*) and B($*z, q*), are:

(4) $$\begin{array}{l} \psi_{\text{A}}=v\left(y\right)w\left(p\right)\\ \psi_{\text{B}}=v\left(z\right)w(q) \end{array}$$

We implemented a logistic choice function with constant noise (Stott, 2006; Chib et al., 2012), so the probability that a subject chooses lottery A is given by:

(5) $$P_{\text{A}}=\frac{1}{1+\exp[-k\left(\psi_{\text{A}}-\psi_{\text{B}}\right)]},$$

where *k* is a parameter that accounts for stochasticity in a subject's choices. A stochasticity parameter *k* = 0 characterizes random choice.

Maximum likelihood estimation was then used to estimate subject-specific distortions in utility and probability for each task. On the *i*th trial, a subject makes a choice *r_i_*. Let *r_i_* = 1 denote choosing lottery A, and let *r_i_* = 0 denote choosing lottery B. Our estimated parameters (α, γ, *k*) for each subject and task maximized the likelihood function over *n* trials:

(6) $$L\left(\alpha ,\;\gamma ,k\right)=\prod_{i\;=\;1}^{n}P_{\text{A}}^{r_{i}}{\left(1-P_{\text{A}}\right)}^{r_{i}}.$$

We used MATLAB's *fminsearch* function with multiple restarts to minimize the negative value of this likelihood function. Because *fminsearch* is a minimization algorithm, we must minimize the negative value of the likelihood in order to find the maximum likelihood estimate. We compared the resulting parameter fits from this risk-sensitive model with those from other potential models, including a risk-neutral model for each subject (with α = γ = 1 and *k* left as a free parameter), a risk-sensitive model using random choices as the input, and a risk-sensitive model with a scaling factor on the standard deviation of movement endpoints (σ' = *c*σ, where *c* is a free parameter) to account for potential distortions in subjects' perceptions of their own motor variability.

As another measure of risk-sensitivity, we computed subjects' frequency of risky choices (fR) in each task. The fR metric is determined as a ratio of the number of trials for which a subject chose the riskier lottery over the safer lottery to the total number of trials in a task. Although this metric does not provide information about risk preferences on individual trials, it provides a global view of risk-seeking (or risk-averse) behavior that we can use to broadly compare across conditions. We performed an additional analysis of how changes in the CPT parameters α and γ would translate to changes in fR (see Section Relation between CPT and fR). Briefly, fR increases with α, so increased valuation of rewards would result in a larger number of risky choices. But the relationship between fR and γ is more complex (and depends on α), so changes in probability weighting would not straightforwardly predict the changes in risky choice behavior.

Increased risk-aversion in movement control could arise from a reduced valuation of the reward associated with completing a movement. This would manifest as a lower α value and a lower fR. Alternatively, risk-aversion could arise from an overweighting of the chance of a movement error that would lead to a fall. This would manifest as a reduced γ value, reflecting an overweighting of small-probability events.

### Skin conductance

We measured changes in skin conductance throughout this experiment using the BIOPAC MP35 acquisition hardware, collecting data at 1000 Hz. Disposable electrodes were placed on the subject's left hand, on the distal phalanx of the index and middle fingers. Skin conductance level (SCL) for each subject was calculated as a percent increase over a baseline condition, during which subjects sat quietly for 5 min. SCL data is available for 19 of the 20 subjects; one subject's SCL data is not presented due to a calibration error.

### Data acquisition

In the ARM training task, optical encoders sample the position of the robot handle at 200 Hz. In the WB training task, the dual-top force platform is comprised of two separate force plates (one for each foot) and records eight analog voltage signals for each plate, which we use to compute three-dimensional forces (*F_x_, F_y_, F_z_*) and moments (*M_x_, M_y_, M_z_*) about the center of each plate at 200 Hz. COP for each plate was calculated relative to the center of the dual-top forceplate, [*C_x_ C_y_*], as [*COP_x_ COP_y_*] = [*C_x_ C_y_*] + [*M_x_ M_y_*]/*F_z_*, where *x* and *y* refer to mediolateral and anteroposterior axes, respectively. We calculate the combined COP as a weighted average of the COP for each plate (Winter et al., 1996).

### Statistics

We used paired *t*-tests to compare SCL between the ARM and WB movements and between the Low and High elevations, as well as to compare movement endpoint variability between elevations for each motor task. Pearson's product-moment correlation coefficient was computed to evaluate the relationships across elevation-based changes in SCL, movement variability, and our risk-sensitivity metrics. We used one-sided paired *t*-tests to examine potential differences in CPT parameter fits and in fR between postural threat conditions, against the alternative hypothesis that mean values at High elevation were lower than those at Low elevation (corresponding to increased risk-aversion with postural threat). We used two-sided paired *t*-tests to examine potential differences in CPT parameter fits and in fR between motor tasks. Permutation testing (with 10,000 permutations) was also employed to compare CPT parameter fits between conditions without making assumptions about the underlying distribution of the samples. We used a one-sided paired *t*-test to compare scaling factors *c* on perceived motor endpoint deviation between tasks, against the *post-hoc* alternative hypothesis *c*_WB Low_ < *c*_ARM Low_. Using the Akaike information criterion (AIC), we compared likelihoods of the full risk-sensitive CPT model (with three free parameters) to those of a risk-neutral model (using *k* as the only free parameter), and we also compared likelihoods of the full risk-sensitive model (computed using actual subject choices) to those of a risk-sensitive model using random choices. We additionally used AIC to compare likelihoods of the full risk-sensitive model (with three free parameters) to those of the risk-sensitive model that included the scaling factor on σ (resulting in four free parameters). For all statistical tests, the significance level was set to 5%. Unless otherwise stated, mean values are presented as mean [± SEM].

## Results

### Overview

Both postural threat and motor task impacted risk-sensitivity. The effect of threat on movement decisions is evident from CPT model fits: increased elevation resulted in greater overweighting of small probabilities for WB movement decisions but did not affect ARM decisions. The effect of motor task on decisions emerges from differences in fR: subjects chose riskier lotteries more often in the WB task than in ARM at low elevation. We believe the disparate risk-sensitivity between motor tasks could manifest from an underestimation of endpoint variability in the WB movement.

### Postural threat increases physiological arousal and decreases endpoint variability

**Figure 3:**
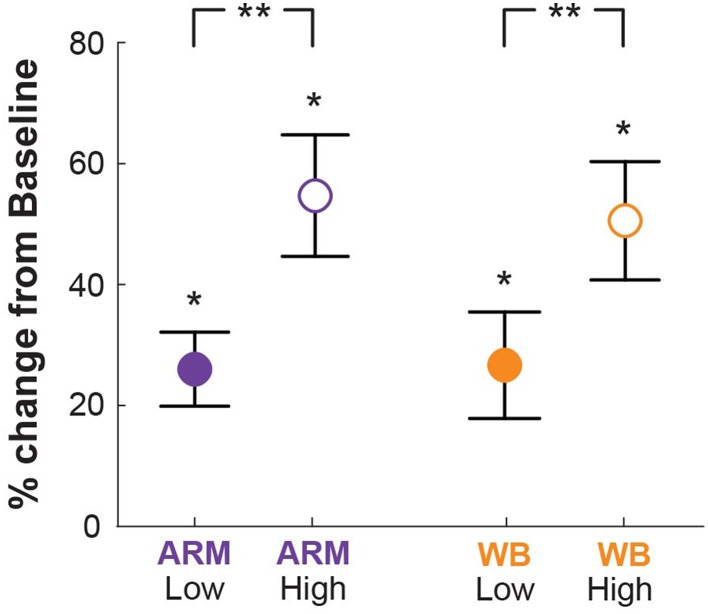
**Skin conductance**. Skin conductance levels (SCL) for all threat conditions relative to Baseline (quiet sitting), wherein 0% indicates no difference from Baseline. SCL at both elevations was significantly higher than at Baseline (^*^p < 0.001), and SCL at the High elevation was significantly higher than at the Low elevation (^**^p < 0.001).

To determine whether the increased elevation led to changes in physiological arousal, we compared SCL across conditions. Mean SCL for the Low and High elevation conditions in each motor task are given in Figure 3. For each condition, SCL was significantly higher than at Baseline (*p* < 0.001). Increasing elevation led to significantly higher skin conductance levels above the baseline condition in both ARM and WB tasks (*p* < 0.001), indicating that subjects responded physiologically to this form of postural threat. There were no significant differences between the two motor tasks within elevation conditions [Low: *t*_(19)_ = 0.48, *p* = 0.73; High: *t*_(19)_ = 2.70, *p* = 0.99].

Paired *t*-tests also show a significant difference in variability between elevation conditions, where σ_High_ is smaller than σ_Low_ for both the ARM task (*p* = 0.032) and the WB task (*p* = 0.034). Individual and mean endpoint variability in each task and condition are provided in the Supplemental information.

We performed a correlation analysis of elevation-based changes in SCL and elevation-based changes in σ. There is a moderate negative correlation between these factors in the WB task (ρ_WB_ = −0.54; *p* = 0.01); that is, subjects who exhibited greater increases in arousal at High elevation also exhibited greater decreases in variability at High elevation. The correlation between SCL and σ in the ARM task is weak and not significant (ρ_ARM_ = −0.25; *p* = 0.39).

### Postural threat affects decisions for whole-body movements, but not for arm-reaching

**Figure 4:**
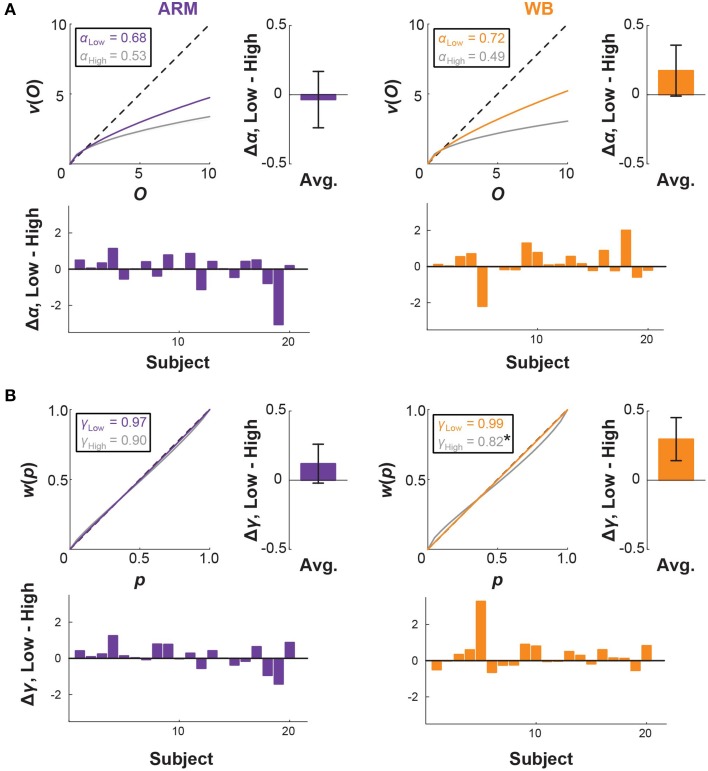
**CPT curves and threat-based changes**. Cumulative prospect theory (CPT) model fits for **(A)** utility and **(B)** probability weighting in the ARM and WB tasks. Curves correspond to median fits for Low elevation (colored) and High elevation (gray). Probability weighting parameter γ is significantly lower in WB High than in WB Low (^*^p = 0.049), suggesting that there is a greater distortion in probability representation under increased threat for the WB task. Bar plots show individual and average differences in each CPT parameter between the Low and High conditions.

Median CPT parameter fits and 95% confidence intervals, taken across all subjects, are given in Table 1 and illustrated in Figure 4. For both ARM and WB, these median fits correspond to exponentially decaying utility (α < 1; ARM Low: 0.68, WB Low: 0.72, ARM High: 0.53, WB High: 0.49) and a tendency to overweight small probabilities in the Low and High elevation conditions (γ < 1; ARM Low: 0.97, WB Low: 0.99, ARM High: 0.90, WB High: 0.82). In accordance with the fourfold pattern of risk attitudes implicated in CPT (Tversky and Kahneman, 1992), greater overweighting of small probabilities corresponds with more risk-seeking behavior for small-probability gains and more risk-averse behavior for small probability losses. In the context of movement control, successful target acquisition can be considered a gain, while movement errors are synonymous with losses. Thus, a concave utility function and the direction of the median probability weighting functions suggests increased risk-aversion toward movement errors at High elevation. Figure 4 also provides individual and mean changes in each parameter between the Low and High elevation conditions.

**Table 1:** **Median CPT parameter fits (all subjects)**.

	**α**	**γ**	***k***
ARM Low	0.68 [0.34, 0.88]	0.97 [0.81, 1.06]	5.23 [3.75, 7.42]
WB Low	0.72 [0.29, 0.96]	0.99 [0.75, 1.06]	4.97 [1.82, 9.03]
ARM High	0.53 [0.29, 0.87]	0.90 [0.67, 1.15]	7.35 [4.08, 19.36]
WB High	0.49 [0.36, 0.92]	0.82^*^ [0.46, 1.06]	4.97 [2.57, 15.21]

Median α, γ, and k values with 95% confidence intervals. Asterisk (

* *) indicates a significant difference from Low elevation within motor task (p < 0.05).

We used permutation testing to construct an empirical distribution of the difference between the median parameters, thereby making no assumptions about the distribution of these parameters. This method revealed that γ values in WB High are significantly smaller than those in WB Low (median value 0.82 at High, compared with 0.99 at Low; *p* = 0.049), which was also supported by a paired *t*-test (*p* = 0.049). There was no significant difference between α or γ parameters for any other comparison between motor tasks or between elevation conditions.

### Frequency of risky choices reveals differences between motor tasks

A comparison of elevation-based changes in SCL with elevation-based changes in fR yielded a moderate positive correlation in the WB task (ρ_WB_ = 0.54, *p* = 0.02), so greater physiological arousal at high elevation was correlated with more risk-seeking behavior. There was no correlation between elevation-based changes in SCL and elevation-based changes in fR for the ARM task (ρ_ARM_ = −0.01, *p* = 0.94), so increased physiological arousal was not related to fR in this motor task.

**Figure 5:**
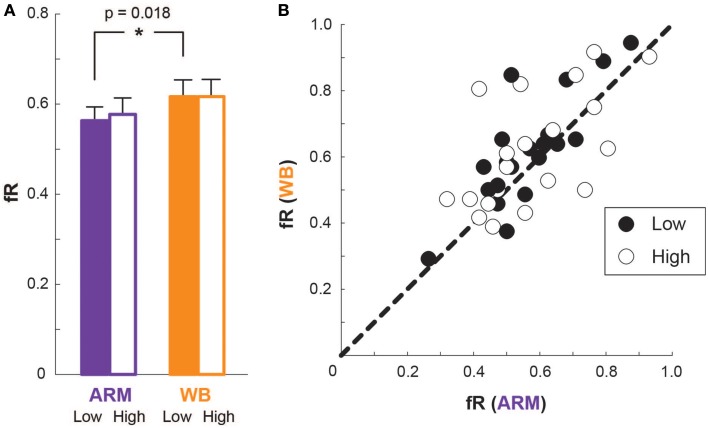
**Frequency of risky choices. (A)** Mean frequency of risky choices (fR) for ARM and WB at Low elevation (filled bars) and at High elevation (outlined bars). **(B)** Each subject's fR in the ARM condition compared with that in the WB condition, at Low elevation (filled circles) and at High elevation (outlined circles). A data point on the line of unity indicates that the subject chose the same number of risky lotteries in both motor tasks.

Figure 5A illustrates average fR for each motor task and elevation. At Low elevation, average fR is greater in the WB task than in the ARM task [*t*_(19)_ = −2.58, *p* = 0.018; ARM Low: 0.56 [0.03], WB Low: 0.62 [0.04]]. At High elevation, however, there is no significant difference in fR between the two tasks [*t*_(19)_ = −1.23, *p* = 0.23; ARM High: 0.58 [0.04], WB High: 0.62 [0.04]].

From Figure 5A, the mean fR at High elevation is greater than (as in ARM) or equal to (as in WB) the mean fR at Low elevation. We should note, however, that an outlier appears to primarily drive this trend. That is, one subject dramatically increased fR going from Low to High elevation in both motor tasks. Upon removing this subject, the fR means are 0.58 [0.03] for ARM Low, 0.63 [0.03] for WB Low, 0.58 [0.04] for ARM High, and 0.60 [0.04] for WB High, making the mean fR at High elevation equal to (as in ARM) or less than (as in WB) the mean fR at Low elevation. So although Figure 5A suggests that mean fR increases slightly, or stays the same, with elevation, removing an outlying subject visually establishes that mean fR actually decreases slightly, or stays the same, with elevation. We repeated all analyses with this outlier subject removed; however, there is no resulting change in our statistical outcomes, nor do our overall findings differ with the exception of the aforementioned trends of mean fR compared between Low and High elevations. Thus, we included the outlying subject for the remainder of the analysis presented here.

A comparison of individual fR values between the two motor tasks are shown in Figure 5B for both Low and High elevations. Here, a data point on the line of unity indicates an identical fR between the two motor tasks at that elevation, while a data point above unity represents someone who was more risk-seeking in the WB task compared with the ARM task, and a data point below unity represents someone who was more risk-seeking in the ARM task compared with the WB task. There is a smaller variance in the difference between ARM and WB fR at Low elevation (σ^2^_fR Low_ = 0.0091) than at High elevation (σ^2^_fR High_ = 0.022). That is, at Low elevation, most subjects have a WB fR that is nearly equal to or greater than their ARM fR, illustrating a relatively consistent increase in risky choices during the WB task compared with the ARM task. But at High elevation, there is a much wider range of differences in individuals' fR between the two motor tasks, with some subjects becoming more risk-seeking in the WB task and others becoming more risk-seeking in the ARM task. This explains why we see a significantly higher fR in the WB task at Low elevation, but there is no significant difference in fR between the paired motor tasks at High elevation.

**Figure 6:**
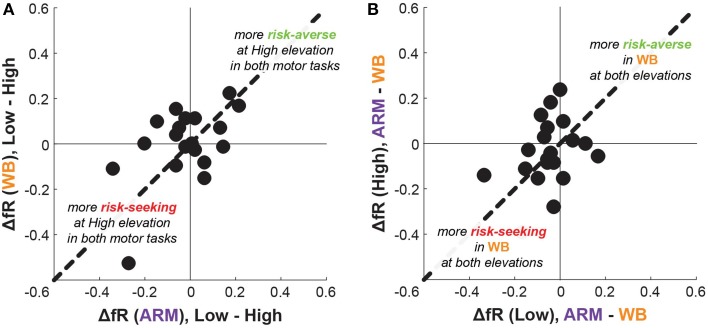
**Difference in frequency of risky choices between elevations and tasks. (A)** Each subject's elevation-based change in fR for the ARM task compared with the WB task. A positive ΔfR corresponds to a subject who had a higher fR at Low elevation than at High elevation, thusly becoming more risk-averse with increasing elevation. A value along the line of unity corresponds to an identical ΔfR in the WB task as in the ARM task, whereas a value above the line of unity—for example—corresponds to having a greater change in risk-sensitivity in the WB task than in the ARM task. **(B)** Each subject's motor-based change in fR for Low elevation compared with the High elevation. A negative ΔfR here corresponds to a subject who had a higher fR in the WB task than in the ARM task. Most points have a negative value on the x-axis, again illustrating that subjects were more risk-seeking in WB than in ARM at Low elevation.

Generally, subjects' risk preferences appeared to change idiosyncratically between movements and threat conditions. This result is further emphasized in Figure 6A, which charts individual differences in fR between Low and High elevation conditions for both motor tasks. While some subjects increased fR (becoming more risk-seeking) in both tasks, and others decreased fR (becoming more risk-averse) in both tasks, still other subjects increased fR in one motor task and decreased it in the other motor task. There is a weak positive correlation in ΔfR between ARM and WB (ρ = 0.29, *p* = 0.23). Figure 6B plots the differences fR between ARM and WB at the Low and High conditions. Here, most points have a negative value on the x-axis, demonstrating that the majority of subjects had a higher fR in WB than in ARM at Low elevation and supporting the findings illustrated in Figure 5.

### Underestimating motor variability increases frequency of risky choices

**Figure 7:**
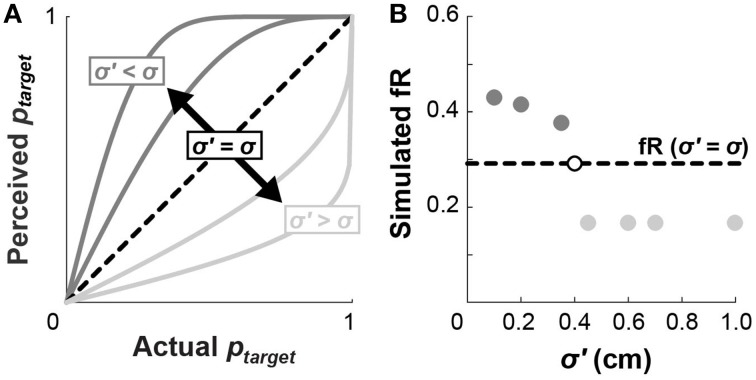
**Perceived motor variability affects fR. (A)** If a subject holds an inaccurate perception of the standard deviation of their endpoints (dark gray: σ′ < σ; or light gray: σ′ > σ), they will have a distorted perception of the probability they will hit a target in accordance with Euation (2). This distortion would effectively alter the lottery probabilities shown in Figure 2A according to their perceived *p*_target_. **(B)** Inaccurate perceptions of σ would hypothetically affect subject choices. Believing yourself to be *more* accurate than you actually are (dark gray: σ′ < σ) would *increase* fR, while believing yourself to be *less* accurate than you actually are (light gray: σ′ > σ) would *decrease* fR. In this example, the simulated subject has σ = 0.40 cm, but the pattern of σ′ affecting fR holds across values of σ.

Perception of motor variability may also influence choice behavior, since we do not explicitly show subjects the probability that they will hit a given target. For example, if subjects believe themselves to be more accurate than they actually are, they would perceive their probabilities of hitting the target to be higher than those listed in Figure 2A. Conversely, if subjects believe themselves to be less accurate than they actually are, they would perceive themselves to have lower probabilities of hitting the targets (Figure 7A). These perceived values of *p_target_*, determined from Equation (2) according to an alternate standard deviation of movement endpoints (σ′ < σ or σ′ < σ), would replace the probabilities used to calculate cumulative prospects in Equation (4).

We next simulated how perceived probabilities, arising from σ′, would affect fR. For each trial, a simulated subject chooses the lottery with a higher expected value, computed using the perceived probabilities. If the selected lottery is also riskier according to the original (undistorted) probabilities, then the number of risky choices increments. Figure 7B compares fR for numerous values of σ′. Underestimating motor variability (σ′ < σ; thinking you are more accurate than you actually are) results in higher fR, whereas overestimating motor variability (σ′ < σ; thinking you are less accurate than you actually are) results in lower fR. This analysis verifies that distorted perceptions of endpoint variability influence choice behavior, and may in part explain why subjects choose riskier lotteries in the WB task.

We ran our CPT model with a scaling factor on σ as an additional free parameter (σ′ = *c*σ). Maximum likelihood estimation fits for this model did not appreciably affect median values of α or γ. The resulting fits for *c* were not significantly different from 1 in any task or elevation condition. In light of the finding that subjects chose risky lotteries more often in the WB task at Low elevation, we also tested the *post-hoc* alternative hypothesis that *c*_WB Low_ < *c*_ARM Low_. The idea that smaller estimates of motor variability lead to increased fR is substantiated by the simulation shown in Figure 7. However, a one-tailed paired *t*-test fails to reject the null hypothesis that there is no difference between *c*_WB Low_ and *c*_ARM Low_ (*p* = 0.74).

### Exploration of alternate CPT models

To examine the fidelity of our parameter fits, we computed the AIC from the maximum likelihood of the model. Preferred models are those with minimum AIC values. We compared the AIC for the risk-sensitive (full) CPT model with three free parameters against a risk-neutral (null) model with α and γ set to unity and with k as the single free parameter. The risk-sensitive model had a lower AIC than the risk-neutral model for all subjects; on average, AIC_full_ = 44.8 and AIC_0_ = 77.5. We also compared the AIC for the risk-sensitive model with actual subject choices against a risk-sensitive model with random choices. We generated 100 sets of random choices for each subject and task and used the maximum likelihood of these sets to calculate an AIC value for the random choice model. The model with actual subject choices had a lower AIC for all subjects than a model with random choices; on average, AIC_rand_ = 109.7. Thus, a risk-sensitive model is better able to describe subjects' choices, and these choices do not appear to be random. Including a scaling factor on σ, to account for potential distortions in perceived motor variability, did not improve our model fits; on average, AIC_*c*σ_ = 46.1. Overall, the 3-parameter risk-sensitive model is simpler while maintaining the lowest average AIC. A summary of AIC values for each task and model type is provided in Table 2.

**Table 2:** **CPT model comparison**.

	**AIC_full_**	**AIC_0_**	**AIC_rand_**	**AIC_*c*σ_**
Parameters	3 (fit α, γ, *k* with subject choices)	1 (α = γ = 1, fit *k* with subject choices)	3 (fit α, γ, *k* with random choices)	4 (fit α, γ, *k, c*σ with subject choices)
ARM Low	47.8	80.6	108.6	47.0
WB Low	43.7	76.3	110.1	47.8
ARM High	43.5	80.5	111.1	44.4
WB High	44.3	72.8	111.6	45.2
Mean	44.8	77.5	109.7	46.1

*AIC values for four iterations of CPT models. The full risk-sensitive model (AIC_full_, with three free parameters) exhibits best performance*.

### Within-subject consistency

On average, subjects made consistent choices in 89.8% of the lotteries for the repeated task, with most discrepancies lying on the diagonal of the outcome-probability matrix (Figure 2A), immediately adjacent to the presented reference choice.

The trends we found using a CPT analysis also hold when we compare the repeated WB Low task with WB High. That is, using this repeated task, there is still less exponential decay in utility at Low compared to that at High, and an overweighting of small probabilities. Importantly, permutation and paired *t*-tests still show that γ is significantly greater in this repeated WB Low than in the original WB High. We also saw no significant difference in fR between the original WB Low and the repeated task (paired *t*-test; *p* = 0.84), and there is still a larger fR for the repeated WB Low task than ARM Low (paired *t*-test; *p* < 0.001).

### Relation between CPT and fR

Median α and γ values are similar between ARM and WB at Low elevation, providing no additional information about consistent distortions that might prompt subjects to choose the risky lottery more often in WB than in ARM, as established by the fR metric. At High elevation, median values are again similar between ARM and WB, but they indicate greater exponential decay of utility compared with Low elevation, as well as greater underweighting of large probabilities.

**Figure 8:**
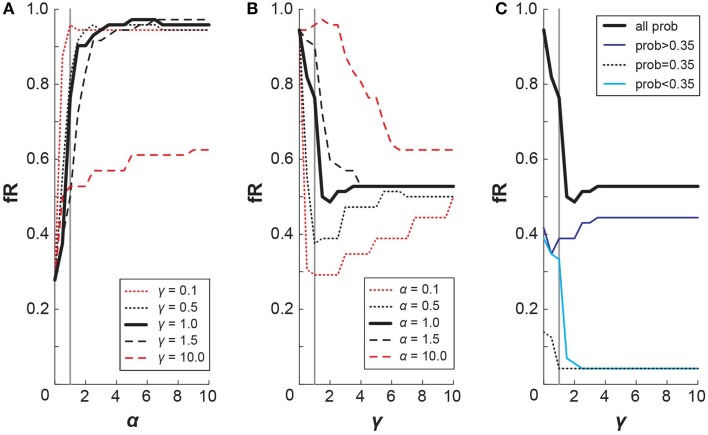
**Mapping CPT onto fR**. Simulated results depicting how changes in CPT parameters affect fR. **(A)** Increases in α generally result in increased fR, and this effect appears to hold irrespective of γ. **(B)** Increases in γ generally result in decreased fR for γ < 1; the overall effect of γ on fR is more complex and varies notably with value of α. **(C)** The probability of the riskier lottery contributes to fR differently in accordance with the shape of the probability weighting function. Relative contributions of small probabilities, the inflection probability, and large probabilities are shown for a simulation with α = 1.

It is pertinent, then, to directly address how our different risk-sensitivity metrics map onto each other. Particularly, how does a decrease in probability weighting, as seen in the WB task, translate to a change in the fR? For each of the lottery pair presented in this experiment, we simulated the choices that subject with certain CPT parameters would make. To do so, we selected α and γ values and calculated the resulting cumulative prospect of the lotteries from Equation (4). When considering each lottery pair, the simulation then chose the lottery with a higher cumulative prospect, which may or may not be the lottery that is riskier. From the simulation's choices, we tallied the number of riskier lotteries chosen, which gave the fR metric for that set of CPT parameters. It follows then that we can examine how small changes in α and γ affect fR (Figure 7). From Figure 8A, we observe that fR generally increases as α increases, and this pattern holds for different values of γ. For α < 1 (exponential decay of utility), smaller γ values produce larger difference in fR. From Figure 8B, we can see that changes in γ have a more complex effect on fR, as fR generally decreases with γ for small γ, but larger γ can result in increased fR. For γ < 1 (underweighting of large probabilities), smaller α values produce larger changes in fR if α ≥ 1. Due to the S-shaped and asymmetric nature of the probability weighting function, the specific probabilities given in the lottery pairs will contribute differently to fR. Figure 8C replicates the mapping of γ onto fR for a fixed α = 1, and it also shows how the value of the riskier lottery's probability contributes to that fR. Thus, changes in γ have competing effects on low and high probability lotteries. Since in this experiment the distribution of low and high probabilities was approximately even, this likely washed out the effect of a change in γ on fR.

Going from Low to High elevation, our subjects exhibited a significant decrease in γ for the WB task (median values: γ_Low_ = 0.99, γ_High_ = 0.82). According to our simulation, this would result in a slightly higher fR for WB High compared with WB Low. Such an effect is not observed in the fR data (as shown in Figure 5 in the main text, or with the outlying subject removed), as it may be washed out by an accompanying decrease in α at High elevation.

## Discussion

Skin conductance measures confirmed that subjects experienced a physiological response to the postural threat presented in this experiment, which was also correlated with reduced movement variability in the WB task. Postural threat and motor task both affected movement-based risk preferences. Increasing postural threat in the form of elevation resulted in greater overweighting of small probabilities in the WB task, which is consistent with increased risk-aversion toward potential movement errors. We also found that individuals are more risk-seeking in WB leaning movements than in ARM at ground level, though this difference in risk preferences between motor tasks cannot be explained solely through consistent distortions in utility or probability weighting.

Irrespective of elevation, the median CPT fits for both motor tasks align overall with risk-seeking behavior for small probability gains and risk-averse behavior for small probability losses, with an exponential decay in utility and an overweighting of small probabilities for both motor tasks. These risk preferences contradict the trends found by Wu et al. (2009, 2011) for a pointing task. When comparing choices in a motor lottery to those in a classical economic lottery, these authors found evidence for underweighting of small probabilities in the motor domain and the typical overweighting of small probabilities in the economic domain. A functional magnetic resonance imaging (fMRI) study (Wu et al., 2011) revealed that subjective utility is encoded in the medial prefrontal cortex (mPFC) and the posterior cingulate cortex (PCC) of the brain. Probability, on the other hand, is represented differently in the mPFC depending on whether is it explicitly presented (as in the economic lottery) or implicitly presented (as in the motor lottery). Our choice tasks implemented implicit probabilities for both motor tasks and elevations, so we do not expect any observed differences in probability weighting to be confounded by the different neural mechanisms of probability encoding.

In the WB task, elevation further distorted probability weighting, with most subjects overweighting small probabilities to a greater extent when standing atop a 0.8 m platform than when standing at ground level. With concave utility, this shape of the weighting function agrees with the fourfold pattern of risk attitudes, describing risk-seeking behavior for small-probability gains and risk-averse behavior for small-probability losses. In the context of goal-directed movement, we interpret gains as target acquisition and losses as movement errors. Greater overweighting of small probabilities, then, indicates that subjects adopted a more cautious strategy at high elevation due to overweighting the probability of errors.

The observed changes in probability weighting appear to align with the findings of Neyedli and Welsh (2014), who noted a preference for probability information over value information when in a motor decision-making task. Participants were offered choices between target configurations for a pointing movement, in which a penalty region overlapped the target. By varying the amount of the point-based penalty (value information) as well as the distance between the target and penalty (probability information), the authors found that participants were more sensitive to probability, preferring configurations with a larger chance of hitting the target to configurations with lower penalty values. If individuals process probability more readily than value during movement selection, it holds that an altered decision strategy would manifest itself more clearly in probability weighting than in utility.

Interestingly, subjects only altered probability weighting in the movement that was more pertinent to the imposed postural threat. Successful completion of the WB motor task required subjects to lean over the edge of the elevated platform, thereby directly confronting them with the increased threat. Previous studies of postural threat have noted changes in postural control when standing or moving at increased elevations—namely, tighter control of the COP along both the anteroposterior and mediolateral axes, posterior shifts in the COP, and reduced displacement and velocity of the COP and center of mass during voluntary movement. These postural control changes have been shown to scale with elevation and are more pronounced at platform heights greater than 1.5 m (Adkin et al., 2000, 2002, 2008; Davis et al., 2009; Cleworth et al., 2012). In our experiment, movement endpoint variability decreased at high elevation for both the ARM and WB tasks. We observe that subjects are reducing the extent of their movements in the mediolateral direction, while the anteroposterier movement extent is fixed to the target distance. Adkin et al. (2000) previously reported a linear decrease in mediolateral variability of the COP with elevation, and we have shown that these results also hold for a forward leaning movement. Moreover, decreased variability is moderately correlated with increased SCL in the WB task. In summary, when performing or planning a movement under threat, individuals will overweight the probability of a fall when a threat is salient to the movement. Concurrently, they tighten control of their posture to reduce motor variability. We anticipate that the observed changes in choice behavior would also scale with elevation, resulting in even greater distortions in probability weighting for the WB task.

We introduced postural threat in this experiment without altering the motor tasks, allowing us to directly probe the effect of emotional stress on movement choice behavior. Even in economic decision making, only recently have systematic investigations of stress effects been performed. Mild psychosocial stress, often induced using the Trier Social Stress Test (TSST), appears to disparately affect gains and losses. For example, in a modified Game of Dice Task, Pabst et al. (2013) found that participants did not alter behavior in gains but made fewer risky decisions in losses after performing the TSST. Such behavior supports the idea that elevation would increase risk-aversion in relation to movement errors. Yet, Porcelli and Delgado (2009) observed an opposite effect, with increased risk-taking for losses as well as decreased risk-taking for gains. Subjects in this experiment were stressed by immersing their dominant hand in an ice-water bath and were asked decide between lotteries of equal expected value and varying probabilities. Unlike in our experiment, both of these studies provided feedback about the outcome after each choice, which may have contributed to their conflicting findings. Buckert et al. (2014) employed the TSST and choices between risky lotteries, did not provide trial-by-trial feedback, and discovered that stress increased risk-taking in the gain domain (linked with cortisol responses) and did not significantly affect risk-taking in losses. All of these stress and decision making studies quantify risk-sensitivity by tallying the number of risky choices, similar to our fR metric. To date, no study to our knowledge has examined the effects of stress on utility and probability weighting. We observed that stress, in the form of elevation-based postural threat, specifically affected decision-making processes by further distorting the probability weighting function.

We had previously assessed risk-sensitivity in movement using a continuous motor task, wherein subjects used out-and-back ARM or WB leaning movements to maneuver a cursor toward the edge of a virtual “cliff” (O'Brien and Ahmed, 2013). They were given a point score for each trial, with higher rewards for traversing closer to the cliff edge and a penalty if the cursor moved beyond the cliff edge. In comparing subject endpoints with a variability-based model of optimal movement planning, we saw that subjects moved closer to the cliff edge than was appropriate for maximizing their expected reward, suggesting risk-seeking behavior. Moreover, subjects were consistently more risk-seeking in the WB task than in arm-reaching. However, it was unclear whether this disparity in risk-sensitivity between the two types movement resulted from differences in utility (subjective valuation of the rewards and penalties), differences in distorted probability weighting (where probability is again tied to motor variability) or some combination thereof. In the present study, we specifically explored potential differences in probability weighting and utility between these movements using a discrete, choice-based paradigm. Subjects chose riskier options more frequently in the context of the WB movement than in ARM at low elevation (ground level), which supports our previous findings. However, from our CPT analysis, there were no significant differences in utility or probability weighting between the two motor tasks at low elevation. Median parameter fits (Figure 4) further illustrate that distortions in utility and probability weighting were indeed very similar between motor tasks within elevations. Another possible explanation for the dissimilar choice behavior between ARM and WB leaning may be in subjects' perceptions of their own motor variability. We demonstrated that underestimating endpoint variability would result in inflated fR (see Section Relation between CPT and fR and Figure 8). Adjusting our risk-sensitive CPT model to include a scaling factor on endpoint deviation did not produce better parameter fits, nor did we find significant differences in the scaling factor between motor tasks. It is likely that a combination of distortions—in utility, probability weighting, and perceptions of motor variability—contribute to the observed choice behavior and effects are difficult to tease out due to between-subject variance. It also remains to be seen whether differences in motor costs or subjective valuation of effort would separately influence risk-sensitivity in the two motor tasks. During training, we controlled for effort by having subjects perform the same movement in each trial. During the decision task, subjects were simply choosing between lotteries, and movement effort was not immediately realized after making each choice.

We maintain that the perceived changes in risk preferences between tasks or conditions results from changes in threat and motor task rather than from inconsistencies in individual subjects' choices. Our subjects repeated one of the motor tasks (WB leaning at low elevation) during the experimental session, and were relatively consistent in their choices between the original task and the repeated task (see Section Within-subject Consistency). Overall, our findings hold whether we examine the original WB task or the repeated task, suggesting that differences between the low and high elevations and between the arm and WB movements are not simply due to within-subject choice fluctuations.

This is the first study to investigate changes in movement risk-sensitivity under increased postural threat. Our findings demonstrate that postural threat does affect risk-sensitivity in movement, and the threat may induce risk-aversion in a salient movement task. Future work may benefit from harnessing fMRI (Wu et al., 2009, 2011) or electroencephalography (EEG) (Pardo-Vazquez et al., 2011, 2014) to examine how threat affects the encoding of sensorimotor decisions.

### Conflict of interest statement

The authors declare that the research was conducted in the absence of any commercial or financial relationships that could be construed as a potential conflict of interest.
