# Supplementary material for: Threat affects risk preferences in movement decision making
Source: Front Behav Neurosci. 2015 Jun 9;9:150. doi: 10.3389/fnbeh.2015.00150 (PMC4460527; doi:10.3389/fnbeh.2015.00150)
Supplement: Supplementary file 1 [file DataSheet1.PDF]

Supplementary Material

Threat affects risk preferences in movement decision making

Megan K. O'Brien [1]*, Alaa A. Ahmed [1]

[1]Neuromechanics Laboratory, Department of Integrative Physiology, University of Colorado, Boulder, Colorado, United States

*** Correspondence:** Megan K. O'Brien, Neuromechanics Laboratory, Department of Integrative Physiology, University of Colorado, 354 UCB, Boulder, Colorado, 80309-0354, United States
megan.obrien@colorado.edu

Keywords: neuroeconomics, sensorimotor control, risk-sensitivity, postural threat, prospect theory.

1. Supplementary Data

1.1. Motor variability

Individual subjects' standard deviation of endpoints, σ, are given in Table S1. Paired t-tests show a significant difference in variability between elevation conditions, where σ_{High} is smaller than σ_{Low} for both the ARM task (t(19)=1.97, p=0.032) or the WB task (t(19)=1.94, p=0.034) (Fig. S1). Decreased mediolateral variability under increased postural threat is not unexpected in the WB task, as Adkin et al. (2000) reported similar findings for quiet standing at higher elevations. Interestingly, we observe similar decreases for seated arm-reaching.

2. Supplementary Figures and Tables

2.1. Supplementary Tables

Supplementary Table 1. Standard deviation of endpoints, σ, in each task and condition (cm).

Subject	ARM Low	ARM High	WB Low	WB High
1	0.51	0.49	0.30	0.33
2	1.58	1.01	0.65	0.65
3	0.54	0.53	0.29	0.21
4	1.00	1.09	0.50	0.48
5	0.87	0.69	0.48	0.43
6	0.66	0.53	0.38	0.28
7	0.78	0.79	0.29	0.35
8	1.72	0.93	0.51	0.42
9	0.58	0.95	0.27	0.27
10	0.98	0.58	0.32	0.35
11	0.75	0.94	0.46	0.62
12	0.91	0.75	0.35	0.39
13	0.99	0.91	0.46	0.59
14	1.05	0.95	0.57	0.37
15	1.10	0.66	0.39	0.38
16	1.05	1.21	0.82	0.39
17	0.87	0.67	0.39	0.30
18	1.08	0.80	0.43	0.34
19	1.27	1.10	0.51	0.26
20	0.58	0.79	0.41	0.27
**Mean**	**0.94**	**0.82**	**0.44**	**0.38**
SEM	*0.21*	*0.18*	*0.10*	*0.09*

2.2. Supplementary Figures

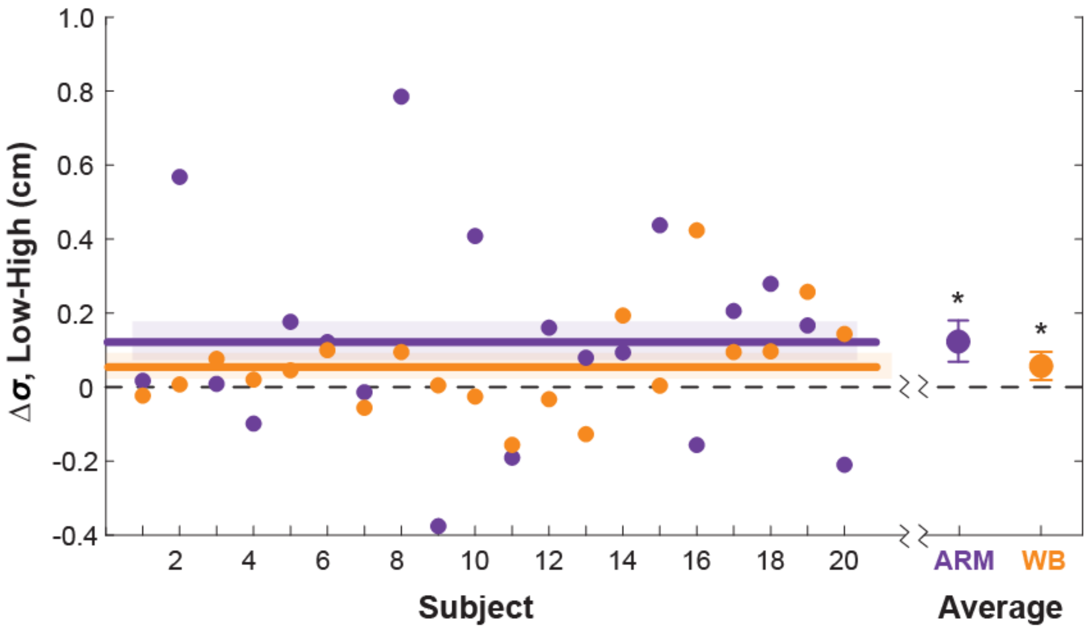

Supplementary Figure 1. Comparison of motor variability between Low and High elevation. Individual (leftward points) and mean (rightward points ± SEM) differences in σ for each motor task. Solid lines and shaded areas are the mean and SEM of each motor task (ARM: purple; WB: orange) projected leftward for ease of comparison with individual data. Positive values depict reduced variability going from Low to High elevation; negative values are increased variability. For both ARM and WB tasks, σ_{Low} is significantly larger than σ_{High} (* $p<0.04$).
